# Crambescin C1 Exerts a Cytoprotective Effect on HepG2 Cells through Metallothionein Induction

**DOI:** 10.3390/md13084633

**Published:** 2015-07-27

**Authors:** María Roel, Juan A. Rubiolo, Eva Ternon, Olivier P. Thomas, Mercedes R. Vieytes, Luis M. Botana

**Affiliations:** 1 Departamento de Farmacología, Facultad de Veterinaria, Universidad de Santiago de Compostela (USC), Campus Lugo, 27002 Lugo, Spain; E-Mails: maria.roel@usc.es (M.R.); ja.rubiolo@usc.es (J.A.R.); 2 Institut de Chimie de Nice, UMR 7272 Université Nice Sophia Antipolis, CNRS, Faculté des Sciences, Parc Valrose, 06108 Nice, France; E-Mails: eva.ternon@unice.fr (E.T.); olivier.thomas@unice.fr (O.P.T.); 3 Institut Méditerranéen de Biodiversité et d’Ecologie Marine et Continentale, UMR 7263 CNRS, IRD, Aix Marseille Université, Avignon Université, Station Marine d’Endoume, Rue de la Batterie des Lions, 13007 Marseille, France; 4 Departamento de Fisiología Animal, Facultad de Veterinaria, Universidad de Santiago de Compostela (USC), Campus Lugo, 27002 Lugo, Spain; E-Mail: mmercedes.rodriguez@usc.es

**Keywords:** crambescin-C1, crambescin-A1, metallothionein, *Crambe crambe*, sponge-derived compounds, transcriptome profiling, antioxidant effect, cell cycle inhibition

## Abstract

The Mediterranean marine sponge *Crambe crambe* is the source of two families of guanidine alkaloids known as crambescins and crambescidins. Some of the biological effects of crambescidins have been previously reported while crambescins have undergone little study. Taking this into account, we performed comparative transcriptome analysis to examine the effect of crambescin-C1 (CC1) on human tumor hepatocarcinoma cells HepG2 followed by validation experiments to confirm its predicted biological activities. We report herein that, while crambescin-A1 has a minor effect on these cells, CC1 protects them against oxidative injury by means of metallothionein induction even at low concentrations. Additionally, at high doses, CC1 arrests the HepG2 cell cycle in G0/G1 and thus inhibits tumor cell proliferation. The findings presented here provide the first detailed approach regarding the different effects of crambescins on tumor cells and provide a basis for future studies on other possible cellular mechanisms related to these bioactivities.

## 1. Introduction

In the past few years the marine environment has proved to be an important source of bioactive natural compounds. Since the fifties, a scientific enthusiasm for these products has emerged due to their extraordinary richness and originality [1,2]. Marine natural compounds are synthesized through the secondary metabolism of marine organisms and are of particular interest for pharmaceutical applications due to their biological activities [1,3,4,5,6,7].

Among marine invertebrates, Porifera (sponges), constitutes one of the most studied phyla owing to their capacity to produce original metabolites [8]. Since the isolation of spongothymidine and spongouridine in 1951 [9], a significant number of sponge secondary metabolites were isolated and identified as antitumoral, anti-inflammatory, antimicrobial, antifungal and antiviral compounds [10]. The noticeable examples of the sponge-derived arabinosilcytosine (Ara-C), currently approved for clinical use in the treatment of acute myeloid leukemia and non-Hodgkin’s lymphoma [11,12], as well as eribulin mesylate, approved by the US Food and Drug Administration for the treatment of metastatic breast cancer; highlight the importance of research on these natural products. Therefore, the increasing interest in the study of their bioactivities is fully understandable.

*Crambe crambe* mainly produces two families of compounds called crambescins and crambescidins [13,14]. Crambescins are mono- or bi-cyclic guanidinic alkaloids firstly isolated from this encrusting Mediterranean sponge [15,16,17].

The available data on the bioactivity of crambescidins indicate that crambescidin 816 (C816) and crambescidin 800 (C800) possess cytotoxic, antifungal, antioxidative, antimicrobial and antiviral activities [18,19,20,21,22,23,24]. C816 also exerts a potent Ca^2+^ antagonist activity, even more intense than nifedipine, a selective blocker of l-type Ca^2+^ channels [14]. Moreover, we have previously evaluated the cytotoxic activity of C816 over several human tumor cell types and characterized some of the cellular mechanisms responsible of the anti-proliferative effect of C816 on human liver-derived tumor cells [24].

While the biological effects of crambescidins have been widely investigated, in the case of crambescins very few data are available. In order to tackle this lack of knowledge and to establish if these compounds could have interest as drugs leads, we examined the effect of crambescin-C1 (CC1) and crambescin-A1 (CA1) on human tumor hepatocarcinoma cells HepG2. According to this, comparative gene expression profiles following CC1 treatment were firstly performed. Obtained results showed that up-regulation of metallothionein mRNA was one of the major cellular responses to CC1. Besides this, effects on cell cycle progression and cellular antioxidant response were also observed. Comparative transcriptome analysis results were then backed up with assays which confirmed the biological effects inferred from them.

## 2. Results and Discussion

### 2.1. CC1 Inhibits Cell Proliferation and Induces Cell Death at High Doses

**Figure 1:**
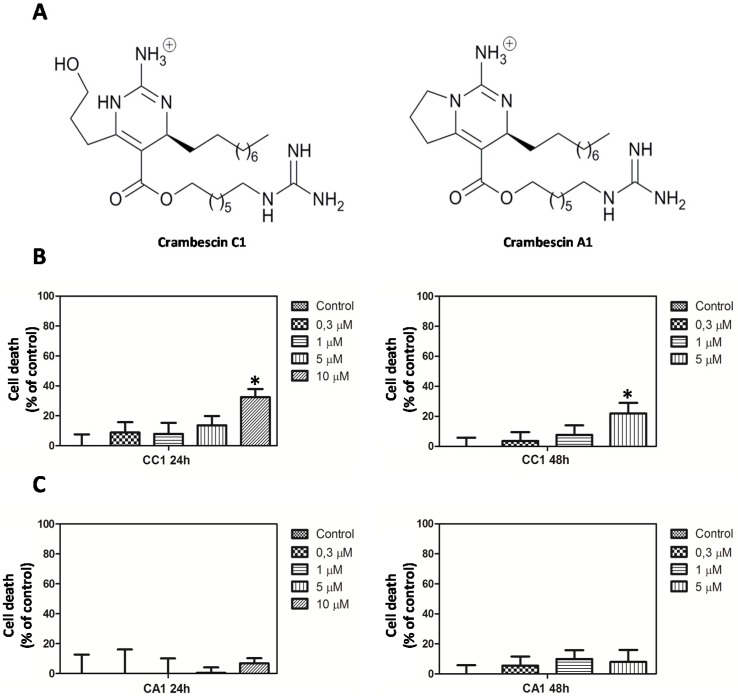
(**A**) Structure of crambescin C1 (CC1) and crambescin A1 (CA1); (**B**) Proliferation of HepG2 cells after CC1 treatment for 24 and 48 h; (**C**) Proliferation of HepG2 cells after CA1 treatment for 24 h and 48 h. In both cases cellular growth was determined by the 3-(4,5-dimethylthiazol-2-l)-2,5-diphenyltetrazolium bromide (MTT) method. * Significant differences respect to controls, *p* < 0.05, *n* = 3.

In order to establish the appropriate concentrations to perform transcriptome analysis, we initially assayed the effects of CC1 and CA1 (Figure 1A) on HepG2 cells growth and viability.

The 3-(4,5-dimethylthiazol-2-l)-2,5-diphenyltetrazolium bromide (MTT) assays showed that after 24 h CC1 reduced cell viability by approximately 33% only at the highest concentration tested (Figure 1B). While no effect was observed after 24 h, an inhibition percentage of 22% was caused by 5 μM CC1 after 48 h (Figure 1B).

Similar doses of CA1 did not reduce cell proliferation whatever the length of the exposure (Figure 1C). Interestingly, CA1’s lack of ability to reduce cellular growth refuted the possibility of a broad crambescin family effect in this regard.

**Figure 2:**
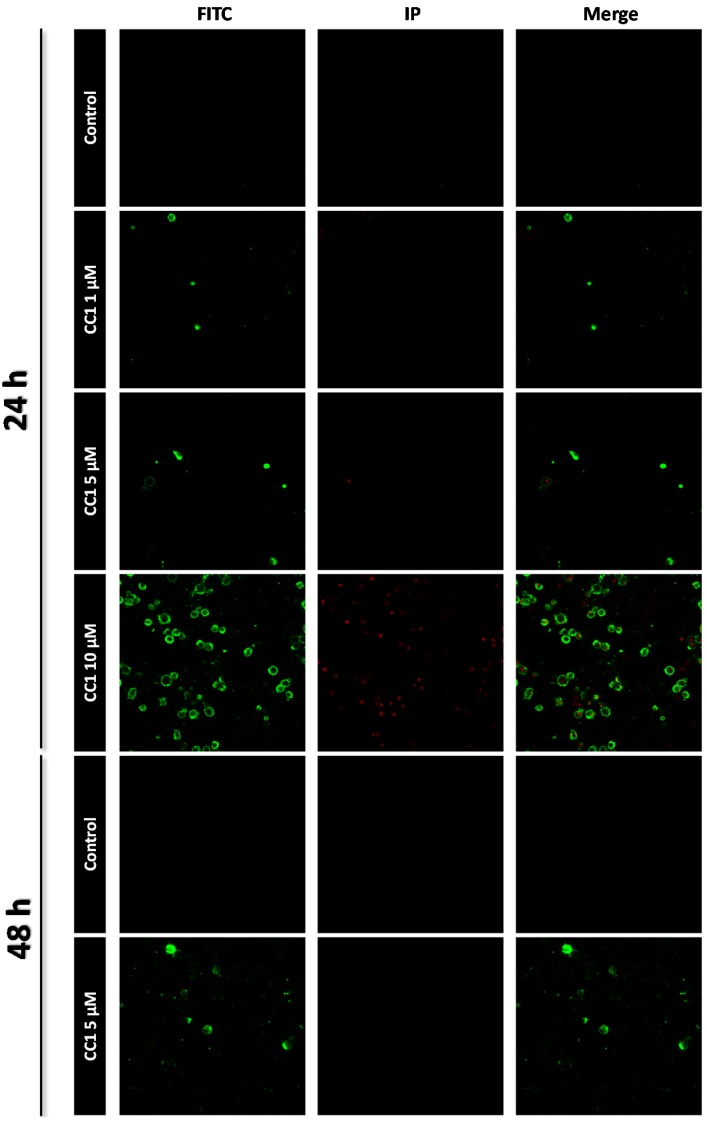
Apoptosis detection by confocal microscopy after 24 h and 48 h treatments with 1, 5 and 10 μM crambescin C1 (CC1). Representative photos of control and treated cells are shown. Fluorescein isothiocyanate (FITC) was used for phosphatidylserine translocation detection (**green**) and propidium iodide (IP) was used for nuclei staining of death cells (**red**).

CC1 induced apoptosis in HepG2 cells as determined by Annexin V and propidium iodide (IP) staining. While no apoptosis was detected after 24 h treatment with 1 μM and 5 μM CC1, 10 μM induced phosphatidylserine translocation. A slight increase of the apoptotic population was also detected after 48 h exposure to 5 μM CC1. Therefore, CC1 induced HepG2 cell apoptosis as a factor of time and dose exposure (Figure 2).

Taking these results into account, just CC1 was selected to perform transcriptome analysis. Concentrations of 1 μM, 5 μM and 10 μM were tested since the highest one induced apoptosis after 24 h. This effect was not observed for 5 μM CC1 but after 48 h. Finally, a non-inhibitory concentration was selected to detect which gene expression variations, if any, were not related to cell death induction.

### 2.2. Transcriptional Alterations Induced by CC1 on HepG2 Cells

**Figure 3:**
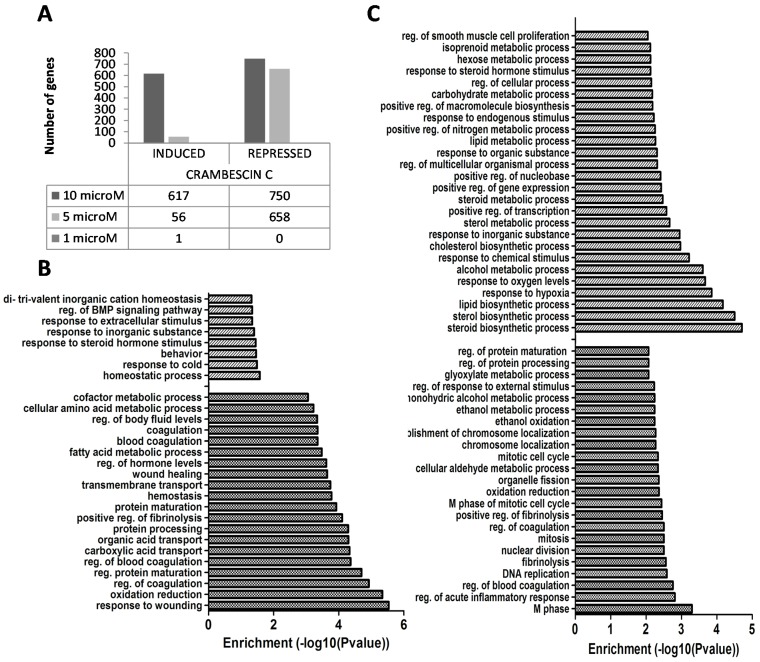
(**A**) Induced and repressed genes after 24 h treatment of HepG2 cells with crambescin C1 (CC1), determined by microarray analysis; (**B**) Biological processes significantly altered in 5 μM CC1 treated HepG2 cells, determined by ontological analysis of up- and down-regulated genes. Dashed bars: up-regulated biological processes. Dotted bars: down-regulated biological processes; (**C**) Biological processes significantly altered in 10 μM CC1 treated HepG2 cells, determined by ontological analysis of up- and down-regulated genes. Dashed bars: up-regulated biological processes. Dotted bars: down-regulated biological processes.

Transcriptomic data analysis showed that, after 24 h, CC1 significantly affected gene expression at 5 μM and 10 μM. These concentrations induced 56 and 617 genes and repressed another 658 and 750 genes respectively (Figure 3A). Gene ontology analysis of up- and down-regulated biological processes showed that 5 μM CC1 repressed genes involved in blood coagulation, transport and metabolism of amino acids and lipids. At the same concentration, CC1 induced genes regulating cell homeostasis and genes implicated in cellular response to extracellular stimulus, inorganic substances and cold (Figure 3B). At 10 μM, CC1 repressed genes involved in cell cycle, coagulation regulation, DNA replication, and oxidation/reduction processes, while inducing genes related to lipid metabolism and gene expression regulation (Figure 3C).

**Figure 4:**
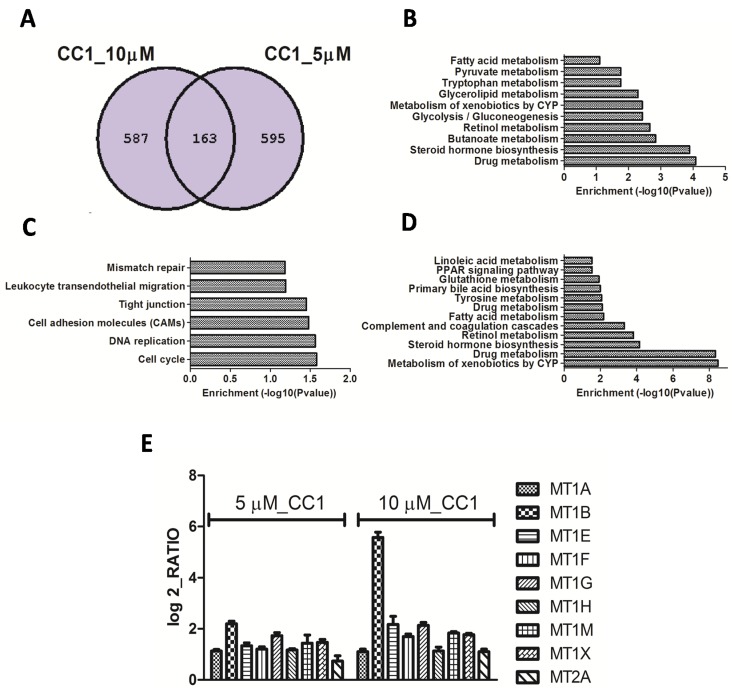
(**A**) Venn diagram for the down-regulated mRNAs in HepG2 cells treated with 5 and 10 μM crambescin C1 (CC1) for 24 h; (**B**) Pathways repressed by CC1 at both concentrations tested, as determined by Kyoto Encyclopedia of Genes and Genomes (KEGGS) pathways; (**C**) Pathways repressed by 10 μM CC1 as determined by KEGGS pathways; (**D**) Pathways repressed by 10 μM CC1 as determined by KEGGS pathways. In all cases an enrichment *p*-value from modified Fisher’s Exact test (EASE Score) <0.05 was selected for significant pathway identification; (**E**) Graph showing the relative increment of metallothionein expression in CC1 treated cultures respect to controls. Shown increments were identified as significant after microarray analysis, *p* < 0.05, *n* = 3.

To compare gene expression patterns of repressed genes, a Venn diagram was used since the lowest concentration induced few genes when compared to the highest one. Both concentrations repressed 163 genes while more than 500 genes were independently repressed by each of them (Figure 4A). According to Keggs pathway analysis, shared genes with the highest enrichment scores are involved in drug and xenobiotic metabolism. Other processes like lipid, retinoid acid, tyrosine, glutathione, linoleic acid, bile acid, and steroid hormone metabolism and/or biosynthesis, although significant, presented lower enrichment scores (Figure 4B). Genes down-regulated by 10 μM CC1, which were not affected in cells treated with 5 μM CC1, are tied to cell cycle control and progression, DNA replication and cellular adhesion (Figure 4C). Genes exclusively down-regulated by 5 μM CC1 are involved in drug, xenobiotic, sugar and lipids metabolisms (Figure 3D). Even though 5 μM CC1 treatment induced few genes, it shared with the 10 μM treatment the induction of metallothioneins (MTs) 1 and 2 (Figure 4E). Therefore, induction of metallothionein expression was a widespread response of HepG2 cells to CC1 exposure.

Metallothioneins are a family of low-molecular weight (6–7 kDa), cysteine-rich intracellular proteins with a well-known metal-binding property [25,26,27]. There are ten human MT isoforms which are jointly encoded by four gene families (MT-1, MT-2, MT-3 and MT-4) grouped at a single locus on chromosome 16q13 [28,29]. The two major isoforms (MT-1 and MT-2) are ubiquitously expressed within tissues while the two minor (MT-3 and MT-4) are mostly found in the central nervous system and stratified squamous epithelia, respectively [30,31,32].

MT-1 and -2 are the most abundant isoforms in human hepatic cells and are also predominant in the human hepatoma cell line HepG2 [33]. Although MT is down-regulated in hepatocellular carcinoma [34], HepG2 cells preserve their capacity to synthesize it in response to diverse stimuli [35,36]. The results presented in this work provide the first evidence that exposure to CC1 causes an up-regulation in MT-1 and MT-2 isoforms expression. MTs levels increase in response to diverse oxidative stress agents, such as glucorticoids, heavy metals and inflammatory signals, indicating that these proteins are tied to pathways involved in cellular response to oxidative damage [37,38,39,40]. In fact, there are two different cysteine clusters within the MT molecule, one closer to the *N*-terminal part designated as β-domain and other closer to the *C*-terminal part known as α-domain. [41,42]. These cysteinyl thiolate groups provide the chemical basis by which MTs can bind metal atoms and function as antioxidants against reactive oxygen and nitrogen species [43].

Cells possess an antioxidant defense system formed by enzymes and other major antioxidants such as reduced glutathione (GSH) [44]. MTs are now understood as part of the defense system since *in vitro* studies confirmed their ability as free radical scavengers [45,46]. Enhanced susceptibility to damage caused by oxidant agents, as for example cadmium or nitric oxide, has been proved in cells with a deficiency in some MTs isoforms [47,48,49]. Similarly, resistance against free radical damage due to MTs overexpression has been documented in cells and tissues [50,51,52]. To determine if the increment of MT expression produced by CC1 affected the redox status of HepG2 cells, we further investigated the effect of this molecule on cells exposed to an oxidant insult.

### 2.3. CC1 Arrests HepG2 Cell Cycle in G0/G1

**Figure 5:**
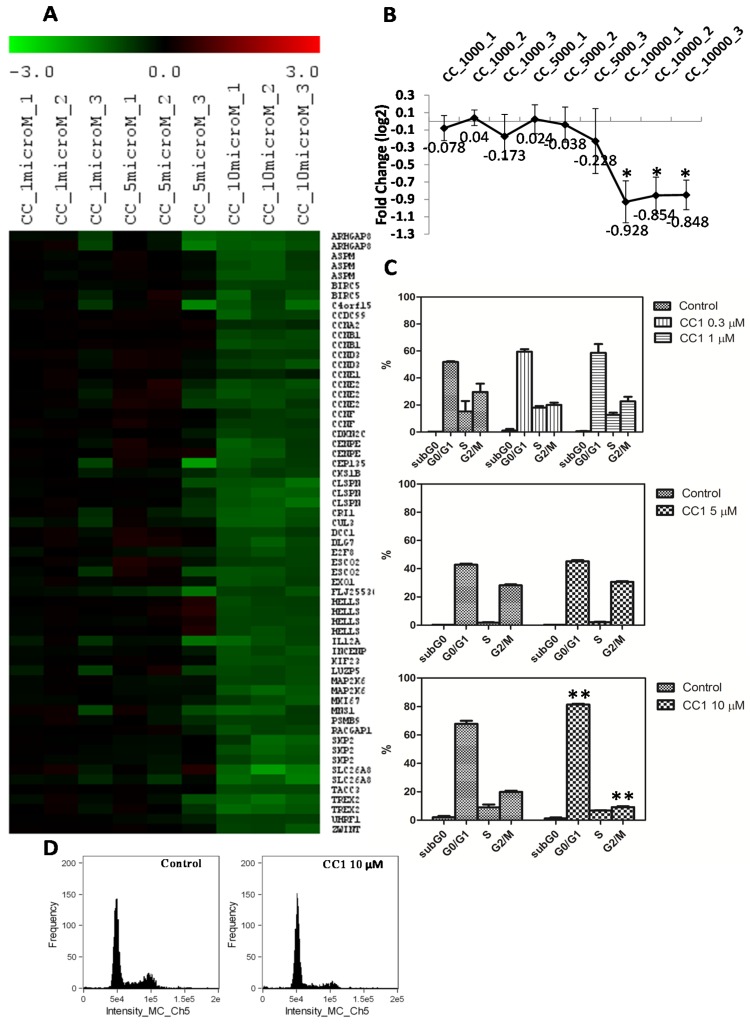
(**A**) Heat map of the differentially expressed (DE) mRNAs coding for proteins involved in cell cycle regulation in HepG2 cells treated with 1 μM, 5 μM and 10 μM crambescin C1 (CC1) with respect to control cells. Green color represents mRNA down-regulation of treated cells with respect to controls; (**B**) Centroid graph for down-regulated genes showed in A. * Significant differences with respect to controls and 5 μM treated cells, *p* < 0.05, *n* = 3; (**C**) Quantification of the cell population percentages in each phase of the cell cycle in control and HepG2 cells treated with 0.3 μM, 1 μM, 5 μM and 10 μM CC1 for 24 h. (*p* < 0.01, *n* = 2); (**D**) Analysis of the relative fluorescence intensity of the stained nuclei. Histograms indicate the differences in the relative proportions of cells in G0/G1 and G2/M phases between control and 10 μM CC1 treated cells.

As initially determined by MTT, CC1 inhibits cell proliferation. Microarrays results showed that CC1 negatively affected the cell cycle progression down-regulating the expression of cyclins A, B, D, and E (Figure 5A,B). According to this, a G0/G1 arrest could be expected.

To confirm this possibility, HepG2 cells were treated with 0.3 μM, 1 μM, 5 μM and 10 μM CC1 for 24 h and analyzed by flow cytometry. CC1 did not cause any significant restriction of the cell cycle progression at 0.3 μM, 1 μM and 5 μM (Figure 5C). However, at 10 μM it produced a significant G0/G1 arrest and decreased the cellular populations in the S and G2/M phases (Figure 5C,D). These results agree with those formerly obtained by the MTT assay on HepG2 cells treated with the same CC1 concentrations for 24 h.

MTT assays showed that, after 48 h, CC1 produced a slight decrease in cell proliferation at 5 μM and had no significant effect at 1 μM. To detect a moderate decrease in cell proliferation it was necessary to increase CC1 concentration until 10 μM for 24 h. Microarray analysis showed that, at this concentration, CC1 negatively affected the cell cycle progression decreasing the expression of cyclins A, B, D, and E. These changes in gene expression are in concordance with the G0/G1 cell cycle arrest detected by flow cytometry after cell treatment with 10 μM CC1 for 24 h [53,54]. Therefore, results demonstrated than the inhibition of the cell cycle progression caused by CC1 ultimately results from changes in the expression of cell cycle-regulatory proteins. Although MTs levels increase does not explain the cell cycle arrest caused by CC1 on HepG2 cells, we cannot exclude their possible implication if we take into account that some studies have suggested the existence of a relationship between MTs and proteins involved in cell cycle control [55,56].

### 2.4. CC1 Protects Cells against Oxidative Injury

**Figure 6:**
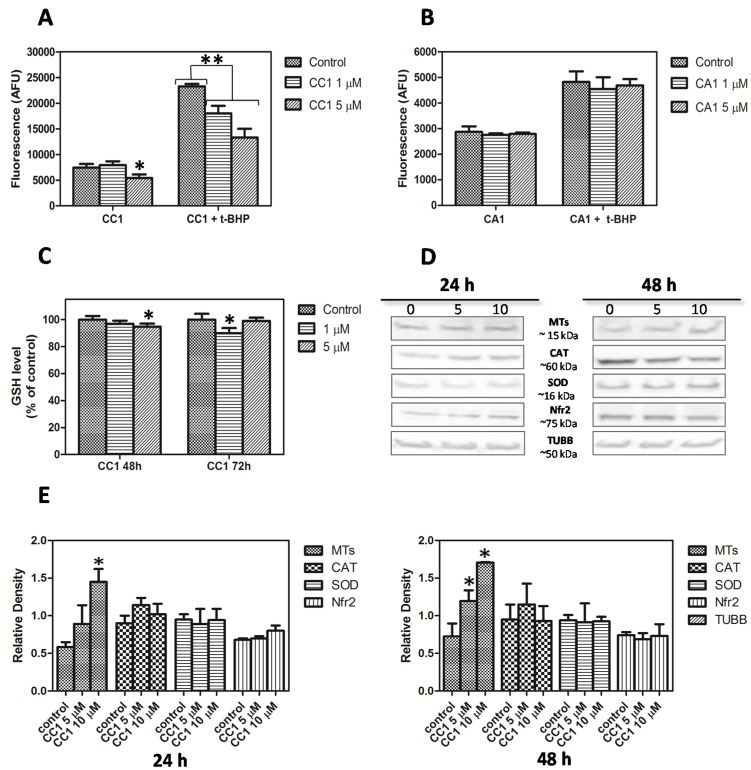
(**A**) Measurement of reactive oxygen species (ROS) generation and evaluation of the HepG2 antioxidant response after 48 h treatment with 1 μM and 5 μM crambescin C1 (CC1); (**B**) Measurement of ROS generation and evaluation of the HepG2 antioxidant response after 48 h treatment with 1 μM and 5 μM CA1; (**C**) Cellular level of reduced glutathione in HepG2 cells treated with 1 μM and 5 μM CC1 for 48 and 72 h; (**D**) Western blot analysis of soluble MT-1 and -2, CAT, SOD, Nfr2 and anti-β-tubulin (TUBB, reference for loading normalization) levels in HepG2 cells treated with 5 μM and 10 μM CC1 for 24 h and 48 h. Representative images are shown. Three experiments were performed for each analyzed protein; (**E**) Quantification of the differences in protein levels among control, 5 μM and 10 μM CC1 treated cells. * Significant differences with respect to controls, *p* < 0.05.

Considering that oxidative stress is a well-known inducer of MTs transcription [57], CC1’s effect on reactive oxygen species (ROS) generation was initially assayed by the 7′,2′-dichlorofluorescein diacetate (DCFH-DA) method. After 48 h exposure, CC1 did not increase ROS production on HepG2 cells at 1 μM and 5 μM (Figure 6A). The same results were obtained for CA1 (Figure 6B).

Because CC1 induced an up-regulation in MTs expression, its capacity to protect cells against oxidative damage was tested. Co-incubations of the natural compound with terbutil-hydroperoxide (*t*-BHP) were done as previously described, and ROS production was measured. CC1 diminished cellular ROS formation caused by *t-*BHP compared with cells just treated with *t-*BHP (Figure 6A). The decreases were significant for all the concentrations tested in a dose-response manner, and the reduction of ROS production was more pronounced at 5 μM. Moreover, significant differences between treatments were detected.

We have demonstrated that CC1 is able to protect cells against *t-*BHP-caused injury while, in the case of CA1, no effect was observed. Results suggest that the up-regulation in MTs expression and the increment of the MT-1, -2 isoforms synthesis induced by CC1 may be strongly linked to the protective antioxidant response caused by this compound after long-term treatments (48 h).

Western blot analysis results obtained after cells treatment with 5 μM CC1 for 48 h confirmed that, at this concentration, CC1 caused a significant increase in MT-1, -2 levels. For the same period of time (48 h) the increments produced by 10 μM CC1 were even higher. At these concentrations significant changes were only detected after 24 h cellular exposure to 10 μM CC1 (Figure 6D,E).

As expected, no antioxidant effect was observed for CA1 (Figure 6B). These results supported those previously obtained with the MTT assays and confirmed that, with regard to its biological activity, CA1 had a minor effect on HepG2 cells. Accordingly, no additional experiment was performed with this compound.

Many cytoprotective enzymes induced in response to oxidative stress are regulated primarily at the transcriptional level [58]; for this reason we carefully analyzed transcriptomic data looking for other alterations related with pathways involved in the cellular antioxidant response. Superoxide dismutase and catalase are two major enzymes in this response; likewise, nuclear factor (erythroid-derived 2)-like 2 (Nrf2) is a transcription factor activated by ROS which controls transcription of genes encoding these detoxification enzymes [59].

Data revealed that, even at 10 μM, no variation was observed in these cellular mechanisms after 24 h exposure to CC1.

Although gene expression of SOD, CAT and Nrf2 was not affected, we performed western blot analysis to rule out protein increases. No variations were detected in relation to control cells after 24 h or 48 h (Figure 6D,E). These results further support the role of MTs in the protection against oxidative stress produced by CC1 on HepG2 cells and point to these proteins as the major agents responsible of this effect.

To further clarify the cause of the antioxidant protection produced by CC1, its influence on GSH levels was investigated. GSH is part of the cellular defense systems against oxidative injury. CC1 produced a slight decrease in GSH levels after 48 h treatment with 5 μM while no effect was observed at 1 μM. Results obtained after 72 h demonstrated that longer exposures to 5 μM CC1 did not cause a major reduction in GSH; conversely, there was a complete reestablishment of GSH to control levels. Finally, when cells were exposed to 1 μM CC1 for 72 h a significant decrease in GSH levels was produced (Figure 6C).

GSH contributes to maintain the thiol/disulfide redox potential in cells by reacting with free radicals. It plays a role during phase II of xenobiotic metabolism and also functions as a glutathione peroxidase substrate [60,61]. We demonstrated that CC1 mildly decreased GSH levels on HepG2 cells after 48 h treatment. The lowest concentration tested (1 μM) did not produce any detectable effect over GSH levels until 72 h, a time at which cells exposed to 5 μM recovered normal GSH concentrations. At these concentrations the increment in MTs synthesis produced in response to CC1 seems to be enough to allow cell survival and retrieval in GSH levels. Previous *in vivo* and *in vitro* studies have documented the existence of a link between MT and GSH levels. In fact, they have demonstrated that MTs induction attenuates the effect of GSH depletors in hepatocytes [62,63]. MTs gene transcription induction in response to oxidative stress has been widely described before, also under conditions that did not reduce cell viability [57,64]. Furthermore, it has been previously established that hepatic levels of MTs are primarily determined at the transcriptional level [65].

Our results demonstrated that CC1 does not increase ROS formation in HepG2 cells, which means that the antioxidant response generated by this molecule is not triggered by ROS production. Additional studies are currently being undertaken to further investigate the mechanism by which this molecule induces MTs and protects cell against oxidative injury.

#### CC1 Increases Nuclear MTs Levels

Cellular MTs distribution pattern was assayed by confocal microscopy. HepG2 cells were treated with low concentrations of CC1 for 12 h in order to ensure the adequate conditions for the detection of the CC1 effect on MTs cellular levels while avoiding any interference with its growth inhibitory ability.

**Figure 7:**
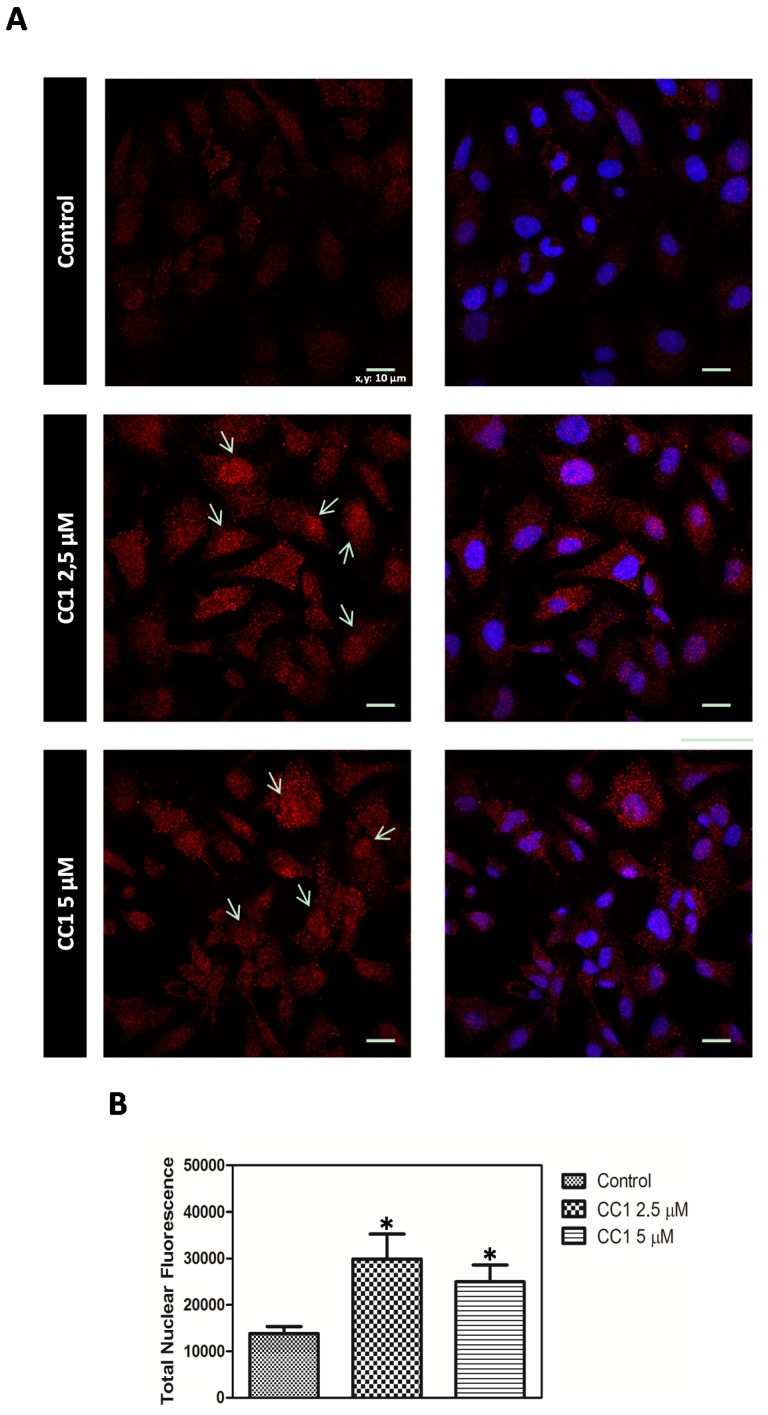
(**A**) MT-1, -2 detection by confocal microscopy in control and HepG2 cells treated with 2.5 μM and 5 μM crambescin C1 (CC1) for 12 h. Representative photos of control and treated cells are shown. Hoechst 33258 was used for nuclei counterstaining (blue) and quantification of nuclear metallothioneins (MTs). Arrows: MTs translocation to the nucleus in treated cells; (**B**) Quantification of the variations caused by CC1 in the levels of nuclear MTs.

Increased nuclear concentrations of MTs proteins were observed after treatments with 2.5 μM and 5 μM CC1 when compared with the levels detected in untreated cells. Therefore, CC1 caused the translocation of MTs from cytoplasm to the nucleus (Figure 7A). To quantify this effect, cellular nuclei stained with Hoechst 33258 were delimitated and MTs fluorescence emission analyzed using the Image J software. Results showed significant increases in nuclear MTs levels in response to 2.5 μM and 5 μM CC1 (Figure 7B).

MTs are mainly cytoplasmic proteins but they can migrate to the nucleus depending on the cell cycle phase or under certain conditions such as cell proliferation and differentiation [66,67,68,69]. Our results demonstrated that even short-term treatments with CC1 caused a cellular translocation of MTs from cytoplasm to the nucleus. MTs are rapidly translocated to the nucleus in response to oxidative stress [70], where they seem to have an antimutagenic role [71]. Apart from ROS, another reactive species has proved to enhance nuclear localization of MTs to protect DNA [39,72].

## 3. Experimental Section

### 3.1. Reagents and Solutions

Eagle’s Minimal Essential Medium (E-MEM), penicillin, streptomycin, anti-cytoplasmic superoxide dismutase, 3-(4,5-dimethylthiazol-2-l)-2,5-diphenyltetrazolium bromide (MTT), anti-catalase, anti-β-tubulin, anti-rabbit and anti-mouse IgG horseradish peroxidase-linked species-specific whole antibodies, CY3-conjugated anti-mouse secondary antibody, Hoechst 33258, propidium iodide, Triton X-100, ethylenediaminetetraacetic acid (EDTA), sodium deoxycholate, sodium dodecyl sulfate (SDS), Tween^®^ 20, sodium chloride (NaCl), trizma base, 7′,2′-dichlorofluorescein diacetate (DCFH-DA), terbutil-hydroperoxide (*t*-BHP) and bovine serum albumin (BSA) were purchased from Sigma-Aldrich (Madrid, Spain). Fetal bovine serum (FBS) was from Cambrex Corporation (Charles City, IA, USA). Aurum™ Total RNA Mini Kit and Precision Plus Protein™ Standards Kaleidoscope™ were obtained from Bio-Rad Laboratories (Barcelona, Spain). The RNA 6000 nano reagents kit was from Agilent Technologies (Madrid, Spain). The cDNA Synthesis System, the NimbleGen One-Color DNA Labeling Kit, the NimbleGen Hybridization Kit, the NimbleGen microarrays, the protease inhibitor complete tablets and the phosphatase inhibitor cocktail tablets were obtained from Roche (Madrid, Spain). Dulbecco’s Phosphate-Buffered Saline (D-PBS), Dulbecco’s Phosphate-Buffered Saline, calcium, magnesium (D-PBS C/M) and ThiolTracker™ Violet were from Gibco^®^, Thermo Fisher Scientific Corporation (Madrid, Spain). Polyvinylidene fluoride (PVDF) membranes and anti-nuclear factor (erythroid-derived 2)-like 2 antibody were purchased from Merck-Millipore (Temecula, CA, USA). SuperSignal^®^ West Pico, SuperSignal^®^ West Femto and anti-metallothionein antibody were from Thermo Fisher Scientific Corporation (Madrid, Spain).

### 3.2. Crambescins Isolation

Both CC1 and CA1 were purified following the protocol proposed by [17] using a specimen of the sponge *C. crambe* sampled in the bay of Villefanche-sur-Mer (Villefanche-sur-Mer, France). The compounds were identified by both ultraperformance liquid chromatography-high resolution mass spectrometry (UPLC-HRMS) and Nuclear Magnetic Resonance spectroscopy (NMR) analysis (95% purity) and were then dissolved in dimethyl sulfoxide (DMSO). The final DMSO concentration for cell treatment was always lower than 0.2%.

### 3.3. Cell Culture

HepG2 cell line was purchased from the American Type Culture Collection (ATCC) and cultured in E-MEM supplemented with 10% fetal bovine serum, 100 UI/mL penicillin and 0.1 mg/mL streptomycin, at 37 °C in a humidified 5% CO_2_ atmosphere.

### 3.4. Cell Viability Assays

Cells were seeded in 96 well plates at a density of 8000 cells/well and incubated overnight before treatment. They were then exposed to different concentrations of CC1 and CA1 for 24 h and 48 h. Viability was determined by the MTT method following the manufacturer’s instructions and absorbance was measured at 570 nm and 670 nm using a Bio-Tek Sinergy plate reader. For each treatment three experiments with *n* = 8 were performed.

### 3.5. Apoptosis Determination

Cells were cultured on poly-lysine coated cover slips at a density of 400,000 cells/well and treated with 1 μM, 5 μM and 10 μM CC1 for 24 h and 5 μM CC1 for 48 h. After washing twice with temperate PBS cover slips were maintained in Annexin binding buffer and stained with Annexin V-FITC and propidium iodide (1%) at room temperature for 15 min in the dark. Two more washes with Annexin binding buffer were made before image acquisition. Stained cells were analyzed with a NIKON-TE2000-3 confocal microscope (Nikon; Amsterdam, Netherlands). Each condition was tested in duplicate and three experiments were done. Representative images of each condition are presented in this work.

### 3.6. Microarrays Assay and Analysis

To obtain RNA for microarray assays, HepG2 cells were treated with CC1 and CA1 for 24 h. RNA from control and treated cells was purified using the Aurum™ Total RNA Mini Kit following the manufacturer’s instructions. Concentration and integrity were determined with a NanoDrop 2000 (Thermo Fisher Scientific) and with a Bioanalyzer 2100 (Agilent) using the RNA 6000 nano reagents kit respectively.

cDNA was synthesized using the cDNA Synthesis System and labeled with the NimbleGen One-Color DNA Labeling Kit following manufacturer’s instructions. Five μg of labeled cDNA from each sample were hybridized onto NimbleGen microarrays (100718_HG18_opt_expr_HX12) using the NimbleGen Hybridization Kit in a NimbleGen HS4 mixer (Roche). After washing and drying, microarrays were scanned with a NimbleGen MS200 scanner (Roche). Scanned images were extracted and bursted using the DEVA 1.2.1 software (Roche). The same software was used for data normalization using robust mass analysis (RMA). For the analysis of differential gene expression, normalized data was loaded in the TM4 Microarray Software Suite [73,74]. Gene lists were analyzed for differentially expressed (DE) genes using the statistic tool SAM (Significance Analysis of Microarrays) and a false discovery rate (FDR) of 5%. Data mining for significant altered metabolic pathways and ontological categories at the biological process level 5 was performed with the DAVID Bioinformatics Database [75,76].

### 3.7. Antioxidant Activity Assay

Free radical formation was quantified using the non-polar compound 7′,2′-dichlorofluorescein diacetate (DCFH-DA). Once deacetylated, this compound is trapped within the cell and oxidized to the fluorescent 7′,2′-dichlorofluorescein (DCF) in the presence of an oxidant. Cells treated with 1 μM and 5 μMT CC1, as well as control cells, were loaded with 20 μM DCFH-DA for 30 min at 37 °C. After that, cells were rinsed twice with fresh medium and 0.1 mM terbutil-hydroperoxide (*t-*BHP) was added to corresponding wells for one hour. Finally, fluorescence intensity was measured using a Bio-Tek Sinergy plate reader with excitation wavelength set at 495 nm and emission wavelength set at 530 nm. Each condition was tested with *n* = 4 and three experiments were performed.

### 3.8. Glutathione Quantification

To estimate the cellular level of reduced glutathione, HepG2 cells were seeded on poly-lysine coated 96 well plates at a density of 8000 cells/well and labeled with ThiolTracker™ Violet dye.

In brief, once cells had been treated with 1 and 5 μM CC1 for 24 and 48 h the incubation medium was removed from the wells and cells were rinsed twice with D-PBS C/M. A pre-warmed 20 μM solution of ThiolTracker™ Violet dye in D-PBS C/M was added to each well and the plates were incubated for 1 h at 37 °C. Finally, cells were rinsed once more with D-PBS and fluorescence was determined at 404 nm excitation and 526 nm emission using a Bio-Tek Sinergy plate reader. For each treatment three experiments with *n* = 4 were carried out.

### 3.9. Western Blot Analysis

Control and CC1 treated cells were re-suspended in RIPA lysis buffer (150 mM NaCl, 1% Triton X-100, 0.5% sodium deoxycholate, 0.1% SDS and 50 mM Tris, pH = 8) and kept on ice for 30 min. Once lysed, cells were centrifuged (15 min at 14,000 rpm, 4 °C) and supernatants were recovered. A Direct Detect™ spectrometer (Merk-Millipore) was used to quantify protein concentration in the lysates and equivalent amounts of protein were resolved by SDS-PAGE and transferred to PVDF membranes.

PVDF membranes were blocked overnight with a solution of 3% non-fat milk and incubated with primary antibodies (anti-cytoplasmic superoxide dismutase (SOD) 1:1000, anti-catalase (CAT) 1:1000, anti-nuclear factor (erythroid-derived 2)-like 2 (Nrf2) 2:1000, anti-β-tubulin (TUBB) 1:5000, anti-metallothionein (MT1-2) 1:1000) for 3 h at room temperature. Then three washed with PBS-0.1% Tween^®^ 20 were made and membranes were incubated again for 1 h at room temperature with secondary anti-rabbit and anti-mouse IgG horseradish peroxidase-linked antibodies. Washed membranes were revealed with SuperSignal^®^ West Pico or SuperSignal^®^ West Femto using a Diversity detector (Syngene; Cambridge, UK). Protein expression was normalized with β-tubulin and differences between treatments were determined with the image analysis software GeneTools (Syngene; Cambridge, United Kingdom).

### 3.10. Cell Cycle Assay by Flow Cytometry

Control and CC1 treated cells were detached from the plates with 0.25% trypsin and 0.2% EDTA and washed twice with 0.1% BSA and 0.3 mM EDTA in PBS. Cells were fixed in cold 70% ethanol. Samples were maintained at 4 °C for 30 min and then washed twice with PBS before staining. After centrifugation, supernatants were discarded and pellets labeled with 50 μL of Telford reagent (75 μM IP, 0.1 mM EDTA, 1.34 mg RNase, 0,1% Triton X-100). Cells were incubated at room temperature for 1 h protected from the light.

An ImageStream^®^ cytometer was used to process the samples and obtained data were analyzed using the IDEAS^®^ Cell Image Analysis software. All conditions were tested in triplicate and for each replicate a total number of 5000 events were acquired. Two experiments were performed.

### 3.11. Confocal Microscopy

HepG2 cells were cultured on poly-lysine coated cover slips and treated with CC1 for 12 h. Treated cells were fixed with 4% paraformaldehyde for 15 min at 4 °C. Then, cells were permeabilized with a solution of 0.2% Triton X-100 in PBS and washed 3 times with PBS-0.1% Tween^®^ 20 before labeling. Cover slips were incubated with an anti-metallothionein-1 and -2 antibody dissolved in a solution of 2% BSA in PBS for 1 h at room temperature. Cells were then washed 3 times and incubated again with 1:500 CY3-conjugated anti-mouse secondary antibody for 1 h protected from light. Before mounting, cover slips were rinsed with PBS-0.1% Tween^®^ 20 another 3 times and 1 μM Hoechst 33258 was added in the last wash for nuclei counterstaining.

Images were obtained with a NIKON TE2000-3 confocal microscope and analyzed using the image processing software Image J. For each treatment representative images were obtained and are presented in this work. 

### 3.12. Statistics

The results were analyzed using the SIGMAPLOT^®^ software. One-way analysis of variance (ANOVA) was used to test for differences among groups and the Holm-Sidak multiple-range test was used for multiple comparisons between groups. A *p* < 0.05 was considered significant.

## 4. Conclusions

The results presented in this work demonstrate that CC1 can induce MTs transcription and synthesis in the human hepatocarcinoma cell line HepG2, protecting cells from oxidative damage at concentrations that do not reduce cell viability. At high doses, it produces a significant decrease in cell viability and also arrests cell cycle progression at the G0/G1 phase. These findings provide the first detailed approach regarding the different bioactivities of crambescins on tumor cells and provide a basis for future studies. We are currently performing further experiments to elucidate the cellular mechanisms underlying these effects.

## Supplementary Files

Supplementary File 1

## References

[1] Riguera R. (1997). Isolating bioactive compounds from marine organisms. J. Mar. Biotechnol..

[2] Leal M.C., Madeira C., Brandão C.A., Puga J., Calado R. (2012). Bioprospecting of marine invertebrates for new natural products—A chemical and zoogeographical perspective. Molecules.

[3] Jha R.K., Zi-rong X. (2004). Biomedical compounds from marine organisms. Mar. Drugs.

[4] Munro M.H.G., Blunt J.W., Dumdei E.J., Hickford S.J.H., Lill R.E., Li S., Battershill C.N., Duckworth A.R. (1999). The discovery and development of marine compounds with pharmaceutical potential. J. Biotechnol..

[5] Sagar S., Kaur M., Minneman K.P. (2010). Antiviral lead compounds from marine sponges. Mar. Drugs.

[6] Haefner B. (2003). Drugs from the deep: Marine natural products as drug candidates. Drug Discov. Today.

[7] Blunt J.W., Copp B.R., Keyzers R.A., Munro M.H.G., Prinsep M.R. (2014). Marine natural products. Nat. Product Rep..

[8] Hu G.-P., Yuan J., Sun L., She Z.-G., Wu J.-H., Lan X.-J., Zhu X., Lin Y.-C., Chen S.-P. (2011). Statistical research on marine natural products based on data obtained between 1985 and 2008. Mar. Drugs.

[9] Bergmann W., Feeney R.J. (1951). Contributions to the study of marine products. XXXII. The nucleosides of sponges 1.1. J. Org. Chem..

[10] Thakur N.L., Müller W.E. (2004). Biotechnological potential of marine sponges. Curr. Sci..

[11] Mayer A., Glaser K.B., Cuevas C., Jacobs R.S., Kem W., Little R.D., McIntosh J.M., Newman D.J., Potts B.C., Shuster D.E. (2010). The odyssey of marine pharmaceuticals: A current pipeline perspective. Trends Pharmacol. Sci..

[12] Huyck T.K., Gradishar W., Manuguid F., Kirkpatrick P. (2011). Eribulin mesylate. Nat. Rev. Drug Discov..

[13] Berlinck R.G.S., Braekman J.C., Daloze D., Bruno I., Riccio R., Rogeau D., Amade P. (1992). Crambines C1 and C2: Two further ichthyotoxic guanidine alkaloids from the sponge *Crambe crambe*. J. Nat. Products.

[14] Berlinck R.G.S., Braekman J.C., Daloze D., Bruno I., Riccio R., Ferri S., Spampinato S., Speroni E. (1993). Polycyclic guanidine alkaloids from the marine sponge *Crambe crambe* and Ca++ channel blocker activity of crambescidin 816. J. Nat. Products.

[15] Laville R.M., Thomas O.P., Berrué F., Marquez D., Vacelet J., Amade P. (2009). Bioactive guanidine alkaloids from two Caribbean marine sponges. J. Nat. Products.

[16] Berlinck R.G.S., Braekman J.C., Daloze D., Hallenga K., Ottinger R., Bruno I., Riccio R. (1990). Two new guanidine alkaloids from the Mediterranean sponge *Crambe crambe*. Tetrahedron Lett..

[17] Bondu S., Genta-Jouve G., Leirόs M., Vale C., Guigonis J.-M., Botana L.M., Thomas O.P. (2012). Additional bioactive guanidine alkaloids from the Mediterranean sponge *Crambe crambe*. RSC Adv..

[18] Buscema M., van de Vyver G. (1985). Cytotoxic rejection of xenografts between marine sponges. J. Exp. Zool..

[19] Olszewski A., Sato K., Aron Z.D., Cohen F., Harris A., McDougall B.R., Robinson W.E., Overman L.E., Weiss G.A. (2004). Guanidine alkaloid analogs as inhibitors of HIV-1 Nef interactions with p53, actin, and p56lck. Proc. Natl. Acad. Sci. USA.

[20] Lazaro J.E., Nitcheu J., Mahmoudi N., Ibana J.A., Mangalindan G.C., Black G.P., Howard-Jones A.G., Moore C.G., Thomas D.A., Mazier D. (2006). Antimalarial activity of crambescidin 800 and synthetic analogues against liver and blood stage of *Plasmodium* sp.. J. Antibiot. (Tokyo).

[21] Suna H., Aoki S., Setiawan A., Kobayashi M. (2007). Crambescidin 800, a pentacyclic guanidine alkaloid, protects a mouse hippocampal cell line against glutamate-induced oxidative stress. J. Nat. Med..

[22] Aoki S., Kong D., Matsui K., Kobayashi M. (2004). Erythroid differentiation in K562 chronic myelogenous cells induced by crambescidin 800, a pentacyclic guanidine alkaloid. Anticancer Res..

[23] Rubiolo J.A., Ternon E., López-Alonso H., Thomas O.P., Vega F.V., Vieytes M.R., Botana L.M. (2013). Crambescidin-816 acts as a fungicidal with more potency than crambescidin-800 and -830, inducing cell cycle arrest, increased cell size and apoptosis in *Saccharomyces cerevisiae*. Mar. Drugs.

[24] Rubiolo J., López-Alonso H., Roel M., Vieytes M., Thomas O., Ternon E., Vega F., Botana L. (2014). Mechanism of cytotoxic action of crambescidin-816 on human liver-derived tumour cells. Br. J. Pharmacol..

[25] Kagi J., Coombs T.L., Overnell J., Webb M. (1981). Synthesis and function of metallothioneins. Nature.

[26] Nath R., Kambadur R., Gulati S., Paliwal V.K., Sharma M. (1988). Molecular aspects, physiological function, and clinical significance of metallothioneins. Crit. Rev. Food Sci. Nutr..

[27] Palmiter R.D. (1998). The elusive function of metallothioneins. Proc. Natl. Acad. Sci. USA.

[28] Karin M., Eddy R.L., Henry W.M., Haley L.L., Byers M.G., Shows T.B. (1984). Human metallothionein genes are clustered on chromosome 16. Proc. Natl. Acad. Sci. USA.

[29] West A., Stallings R., Hildebrand C., Chiu R., Karin M., Richards R. (1990). Human metallothionein genes: Structure of the functional locus at 16q13. Genomics.

[30] Palmiter R.D., Findley S.D., Whitmore T.E., Durnam D.M. (1992). MT-III, a brain-specific member of the metallothionein gene family. Proc. Natl. Acad. Sci. USA.

[31] Quaife C.J., Findley S.D., Erickson J.C., Froelick G.J., Kelly E.J., Zambrowicz B.P., Palmiter R.D. (1994). Induction of a new metallothionein isoform (MT-IV) occurs during differentiation of stratified squamous epithelia. Biochemistry.

[32] Haq F., Mahoney M., Koropatnick J. (2003). Signaling events for metallothionein induction. Mutat. Res..

[33] Sadhu C., Gedamu L. (1988). Regulation of human metallothionein (MT) genes. Differential expression of MTI-F, MTI-G, and MTII-A genes in the hepatoblastoma cell line (HepG2). J. Biol. Chem..

[34] Tao X., Zheng J.M., Xu A.M., Chen X.F., Zhang S.H. (2007). Downregulated expression of metallothionein and its clinicopathological significance in hepatocellular carcinoma. Hepatol. Res..

[35] Sadhu C., Gedamu L. (1989). Metal-specific posttranscriptional control of human metallothionein genes. Mol. Cell. Biol..

[36] Jahroudi N., Foster R., Price-Haughey J., Beitel G., Gedamu L. (1990). Cell-type specific and differential regulation of the human metallothionein genes. Correlation with DNA methylation and chromatin structure. J. Biol. Chem..

[37] Bauman J., Liu J., Liu Y., Klaassen C. (1991). Increase in metallothionein produced by chemicals that induce oxidative stress. Toxicol. Appl. Pharmacol..

[38] Bauman J.W., Madhu C., McKim J.M., Liu Y., Klaassen C.D. (1992). Induction of hepatic metallothionein by paraquat. Toxicol. Appl. Pharmacol..

[39] Schwarz M.A., Lazo J.S., Yalowich J.C., Allen W.P., Whitmore M., Bergonia H.A., Tzeng E., Billiar T.R., Robbins P.D., Lancaster J.R. (1995). Metallothionein protects against the cytotoxic and DNA-damaging effects of nitric oxide. Proc. Natl. Acad. Sci. USA.

[40] Yamasaki M., Nomura T., Sato F., Mimata H. (2007). Metallothionein is up-regulated under hypoxia and promotes the survival of human prostate cancer cells. Oncol. Rep..

[41] Robbins A., Stout C. (1991). X-ray structure of metallothionein. Methods Enzymol..

[42] Otvos J.D., Armitage I.M. (1980). Structure of the metal clusters in rabbit liver metallothionein. Proc. Natl. Acad. Sci. USA.

[43] Maret W., Vallee B.L. (1998). Thiolate ligands in metallothionein confer redox activity on zinc clusters. Proc. Natl. Acad. Sci. USA.

[44] Janssen Y., Van Houten B., Borm P., Mossman B. (1993). Cell and tissue responses to oxidative damage. Lab. Investig..

[45] Cai L., Klein J.B., Kang Y.J. (2000). Metallothionein inhibits peroxynitrite-induced DNA and lipoprotein damage. J. Biol. Chem..

[46] Kumari M.R., Hiramatsu M., Ebadi M. (1998). Free radical scavenging actions of metallothionein isoforms I and II. Free Radic. Res..

[47] Kondo Y., Rusnak J.M., Hoyt D.G., Settineri C.E., Pitt B.R., Lazo J.S. (1997). Enhanced apoptosis in metallothionein null cells. Mol. Pharmacol..

[48] Lazo J.S., Kondo Y., Dellapiazza D., Michalska A.E., Choo K.A., Pitt B.R. (1995). Enhanced sensitivity to oxidative stress in cultured embryonic cells from transgenic mice deficient in metallothionein I and II genes. J. Biol. Chem..

[49] Zheng H., Liu J., Liu Y., Klaassen C.D. (1996). Hepatocytes from metallothionein-I and II knock-out mice are sensitive to cadmium- and *tert*-butylhydroperoxide-induced cytotoxicity. Toxicol. Lett..

[50] Lazo J.S., Kuo S.-M., Woo E.S., Pitt B.R. (1998). The protein thiol metallothionein as an antioxidant and protectant against antineoplastic drugs. Chem. Biol. Interact..

[51] Ebadi M., Leuschen M., El Refaey H., Hamada F., Rojas P. (1996). The antioxidant properties of zinc and metallothionein. Neurochem. Int..

[52] Oshima Y., Fujio Y., Nakanishi T., Itoh N., Yamamoto Y., Negoro S., Tanaka K., Kishimoto T., Kawase I., Azuma J. (2005). STAT3 mediates cardioprotection against ischemia/reperfusion injury through metallothionein induction in the heart. Cardiovasc. Res..

[53] Koff A., Giordano A., Desai D., Yamashita K., Harper J.W., Elledge S., Nishimoto T., Morgan D.O., Franza B.R., Roberts J.M. (1992). Formation and activation of a cyclin E-cdk2 complex during the G1 phase of the human cell cycle. Science.

[54] Bates S., Bonetta L., MacAllan D., Parry D., Holder A., Dickson C., Peters G. (1994). CDK6 (PLSTIRE) and CDK4 (PSK-J3) are a distinct subset of the cyclin-dependent kinases that associate with cyclin D1. Oncogene.

[55] Méplan C., Richard M.-J., Hainaut P. (2000). Metalloregulation of the tumor suppressor protein p53: Zinc mediates the renaturation of p53 after exposure to metal chelators *in vitro* and in intact cells. Oncogene.

[56] Fan L., Cherian M. (2002). Potential role of p53 on metallothionein induction in human epithelial breast cancer cells. Br. J. Cancer.

[57] Andrews G.K. (2000). Regulation of metallothionein gene expression by oxidative stress and metal ions. Biochem. Pharmacol..

[58] Nguyen T., Sherratt P.J., Pickett C.B. (2003). Regulatory mechanisms controlling gene expression mediated by the antioxidant response element. Annu. Rev. Pharmacol. Toxicol..

[59] Nguyen T., Nioi P., Pickett C.B. (2009). The Nrf2-antioxidant response element signaling pathway and its activation by oxidative stress. J. Biol. Chem..

[60] Viña J., Sáez G., Viña J. (1989). The physiological functions of glutathione. Handbook of Free Radicals and Antioxidants in Biomedicine.

[61] Sies H. (1999). Glutathione and its role in cellular functions. Free Radic. Biol. Med..

[62] Haïdara K., Moffatt P., Denizeau F. (1999). Metallothionein induction attenuates the effects of glutathione depletors in rat hepatocytes. Toxicol. Sci..

[63] Chan H.M., George Cherian M. (1992). Protective roles of metallothionein and glutathione in hepatotoxicity of cadmium. Toxicology.

[64] Samson S.L.-A., Gedamu L. (1997). Molecular analyses of metallothionein gene regulation. Prog. Nucleic Acid Res. Mol. Biol..

[65] Zalups R.K., Koropatnick J. (2000). Temporal changes in metallothionein gene transcription in rat kidney and liver: Relationship to content of mercury and metallothionein protein. J. Pharmacol. Exp. Ther..

[66] Nagel W.W., Vallee B.L. (1995). Cell cycle regulation of metallothionein in human colonic cancer cells. Proc. Natl. Acad. Sci. USA.

[67] Ghoshal K., Jacob S.T. (2000). Regulation of metallothionein gene expression. Prog. Nucleic Acid Res. Mol. Biol..

[68] Cherian M., Apostolova M. (2000). Nuclear localization of metallothionein during cell proliferation and differentiation. Cell. Mol. Biol. (Noisy-le-grand).

[69] Cherian M.G. (1994). The significance of the nuclear and cytoplasmic localization of metallothionein in human liver and tumor cells. Environ. Health Perspect..

[70] Yukihisa T., Yasumitsu O., Kenji I., Kazuo T.S. (2004). Role of metallothionein in the cell cycle: Protection against the retardation of cell proliferation by endogenous reactive oxygen species. J. Health Sci..

[71] Woo E.S., Lazo J.S. (1997). Nucleocytoplasmic functionality of metallothionein. Cancer Res..

[72] Ogra Y., Onishi S., Kajiwara A., Hara A., Suzuki K.T. (2008). Enhancement of nuclear localization of metallothionein by nitric oxide. J. Health Sci..

[73] Saeed A.I., Bhagabati N.K., Braisted J.C., Liang W., Sharov V., Howe E.A., Li J., Thiagarajan M., White J.A., Quackenbush J. (2006). TM4 microarray software suite. Methods Enzymol..

[74] Saeed A., Sharov V., White J., Li J., Liang W., Bhagabati N., Braisted J., Klapa M., Currier T., Thiagarajan M. (2003). TM4: A free, open-source system for microarray data management and analysis. Biotechniques.

[75] Huang da W., Sherman B.T., Lempicki R.A. (2009). Bioinformatics enrichment tools: Paths toward the comprehensive functional analysis of large gene lists. Nucleic Acids Res..

[76] Huang da W., Sherman B.T., Lempicki R.A. (2009). Systematic and integrative analysis of large gene lists using DAVID bioinformatics resources. Nat. Protoc..

