# Supplementary material for: Crambescin C1 Exerts a Cytoprotective Effect on HepG2 Cells through Metallothionein Induction
Source: Mar Drugs. 2015 Jul 27;13(8):4633–53. doi: 10.3390/md13084633 (PMC4556997; doi:10.3390/md13084633)
Supplement: Supplementary File 1 [file marinedrugs-13-04633-s001.docx]

**Supplementary Information**

**Table S1. Biological processes affected in HepG2 cells after 5 µM crambescin-C1 (CC1) treatment, as determined by ontology analysis**.

| **Repressed Genes after 24 h of Treatment with 5 μM CC1** | | | | | |
| --- | --- | --- | --- | --- | --- |
| **Category** | **Term** | **Count** | **%** | ***p* Value** | **Genes** |
| GOTERM_BP_FAT | GO:0009611—response to wounding | 35 | 7.143 | 2.89E-06 | NM_002209, NM_000242, NM_000505, BC016755, BC060039, X04385, NM_013230, NM_139208, NM_000066, AF268691, BX248290, NM_053013, NM_000821, NM_003873, NM_178570, BC050309, NM_005810, NM_052968, NM_000577, NM_000499, NM_002507, NM_000072, BC017249, AY310359, NM_006691, NM_000128, BC069338, NM_005577, NM_000552, NM_014880, NM_004171, NM_000211, NM_001024628, NM_006329, NM_012385, NM_000562, NM_000301, BC007580, NM_002133, CR603101, NM_000362, NM_000893, NM_001994, NM_001976, BC101787, NM_000892 |
| GOTERM_BP_FAT | GO:0055114—oxidation reduction | 39 | 7.959 | 4.57E-06 | NM_144704, NM_153486, AK126917, NM_007101, BC040351, BC074738, BC074771, BC067436, AK092439, NM_000787, AB011157, BC101631, BC101777, AY310359, AK094844, NM_001966, NM_022820, BC009581, BC050378, NM_004051, BC047902, BC100982, NM_001017916, NM_017781, NM_021232, BC015726, NM_001003684, NM_153824, NM_022568, NM_000792, BC000021, NM_013387, NM_000382, BC033862, NM_024955, NM_002153, BC063388, NM_017460, NM_005276, NM_000669, NM_022746, BC069418, NM_152899, NM_030579, NM_000667, NM_000765, NM_000860, BC067419, NM_000499, AF280111, AF280112, NM_000670, NM_018068, AF280113, AF095737, AF095736, BC022319, NM_001609, BC111751, NM_000780, NM_001039716, NM_005989, NM_000672, NM_002084, NM_004549, NM_000777, BC107170, AK000366, NM_000110, NM_002133, BC005844, NM_006907, BC002976, AK058142, BC011964, BC039065 |
| GOTERM_BP_FAT | GO:0050818—regulation of coagulation | 9 | 1.837 | 1.19E-05 | NM_000505, NM_000301, NM_153612, BC060039, CR605456, NM_004616, NM_000128, NM_000893, NM_153609, NM_000892 |
| GOTERM_BP_FAT | GO:0051605—protein maturation by peptide bond cleavage | 12 | 2.449 | 1.99E-05 | NM_000505, NM_000242, BC016755, NM_003020, NM_139208, NM_033280, NM_000066, NM_000562, NM_022034, NM_000128, BC069338, BC009703, NM_000892 |
| GOTERM_BP_FAT | GO:0030193—regulation of  blood coagulation | 8 | 1.633 | 4.32E-05 | NM_000505, NM_000301, BC060039, CR605456, NM_004616, NM_000128, NM_000893, NM_153609, NM_000892 |
| GOTERM_BP_FAT | GO:0046942—carboxylic acid transport | 15 | 3.061 | 4.64E-05 | NM_002614, NM_004171, AF191687, NM_018018, NM_022042, NM_001011554, NM_001024845, NM_000030, NM_020980, NM_005847, NM_173512, AK000846, BC069799, BC026258, BC074724, NM_033518, AK001053, BC035966, BC069819, NM_000072, NM_003044, NM_019849 |

**Table S1.** *Cont.*

| **Repressed Genes after 24 h of Treatment with 5 μM CC1** | | | | | |
| --- | --- | --- | --- | --- | --- |
| **Category** | **Term** | **Count** | **%** | ***p* Value** | **Genes** |
| GOTERM_BP_FAT | GO:0015849—organic acid transport | 15 | 3.061 | 5.00E-05 | NM_002614, NM_004171, AF191687, NM_018018, NM_022042, NM_001011554, NM_001024845, NM_000030, NM_020980, NM_005847, NM_173512, AK000846, BC069799, BC026258, BC074724, NM_033518, AK001053, BC035966, BC069819, NM_000072, NM_003044, NM_019849 |
| GOTERM_BP_FAT | GO:0016485—protein processing | 13 | 2.653 | 5.11E-05 | NM_000505, NM_000242, BC016755, NM_003020, NM_033292, NM_139208, NM_033280, NM_000066, NM_033295, NM_033293, NM_000562, NM_022034, NM_000128, BC069338, BC009703, NM_001223, NM_000892 |
| GOTERM_BP_FAT | GO:0051919—positive regulation  of fibrinolysis | 4 | 0.816 | 7.76E-05 | NM_000505, NM_000301, NM_000128, NM_000892 |
| GOTERM_BP_FAT | GO:0051604—protein maturation | 13 | 2.653 | 1.18E-04 | NM_000505, NM_000242, BC016755, NM_003020, NM_033292, NM_139208, NM_033280, NM_000066, NM_033295, NM_033293, NM_000562, NM_022034, NM_000128, BC069338, BC009703, NM_001223, NM_000892 |
| GOTERM_BP_FAT | GO:0007599—hemostasis | 12 | 2.449 | 1.65E-04 | NM_000552, NM_000505, BC060039, X04385, BX248290, NM_000821, NM_006329, NM_006163, NM_000301, NM_000072, BC005044, NM_000893, NM_000128, NM_001994, NM_005577, NM_000892 |
| GOTERM_BP_FAT | GO:0055085—transmembrane transport | 32 | 6.531 | 1.80E-04 | NM_002614, NM_022042, NM_139177, BC104822, NM_198378, BC101516, BC022379, NM_080866, NM_005835, NM_020980, NM_020342, BC101314, NM_021007, NM_020407, AF521885, BC104857, NM_017767, AB093548, BC036078, NM_006672, NM_130849, NM_145282, NM_178526, BC105995, NM_001011554, NM_021977, BC036869, BC105103, AK074246, NM_005847, NM_198387, NM_001171, BC026258, NM_199352, BC060041, BC033805, BC035966, NM_173596, NM_001652, NM_006920, NM_000583, NM_000443, NM_001039752, XM_370997, NM_182556 |
| GOTERM_BP_FAT | GO:0042060—wound healing | 16 | 3.265 | 2.24E-04 | NM_000552, NM_000505, BC060039, X04385, NM_053013, BX248290, NM_000821, NM_006329, NM_052968, BC007580, NM_000301, NM_002133, CR603101, NM_000072, BC017249, NM_000362, NM_000128, NM_000893, BC101787, NM_001976, NM_001994, NM_005577, NM_000892 |

**Table S1.** *Cont.*

| **Repressed Genes after 24 h of Treatment with 5 μM CC1** | | | | | |
| --- | --- | --- | --- | --- | --- |
| **Category** | **Term** | **Count** | **%** | ***p* Value** | **Genes** |
| GOTERM_BP_FAT | GO:0010817—regulation of  hormone levels | 14 | 2.857 | 2.39E-04 | NM_178471, NM_005420, NM_003020, NM_001039716, NM_007023, AJ409094, NM_005989, BC095502, NM_000349, NM_022568, BC039902, BC107170, NM_000792, BC010550, NM_000499, NM_000670, CR605456, BC063388, AY310359, BC057829, BC022319, AK058142 |
| GOTERM_BP_FAT | GO:0006631—fatty acid  metabolic process | 16 | 3.265 | 3.31E-04 | NM_025149, NM_001009185, NM_005622, NM_001609, CR606981, NM_012254, BC030783, NM_020918, NM_000236, NM_052956, BC068516, NM_182617, NM_000860, CR605456, BC058287, BC029792, NM_024330, NM_014495, NM_001966, NM_001010845, NM_017888 |
| GOTERM_BP_FAT | GO:0007596—blood coagulation | 11 | 2.245 | 4.43E-04 | NM_000552, NM_000505, BC060039, X04385, BX248290, NM_000821, NM_006329, NM_000301, NM_000072, NM_000893, NM_000128, NM_001994, NM_000892, NM_005577 |
| GOTERM_BP_FAT | GO:0050817—coagulation | 11 | 2.245 | 4.43E-04 | NM_000552, NM_000505, BC060039, X04385, BX248290, NM_000821, NM_006329, NM_000301, NM_000072, NM_000893, NM_000128, NM_001994, NM_000892, NM_005577 |
| GOTERM_BP_FAT | GO:0050878—regulation of body fluid levels | 13 | 2.653 | 4.57E-04 | NM_000552, NM_000505, BC060039, X04385, BX248290, NM_000821, NM_006329, NM_006163, NM_000301, NM_000072, CR605456, BC005044, NM_000893, NM_000128, NM_001994, NM_005577, NM_000892 |
| GOTERM_BP_FAT | GO:0051241—negative regulation of multicellular organismal process | 14 | 2.857 | 5.32E-04 | NM_022437, NM_000505, NM_153612, BC060039, NM_000040, NM_013230, NM_004616, BC050309, NM_002507, NM_000301, NM_002133, CR605456, NM_000128, NM_000893, NM_153609, NM_000892 |
| GOTERM_BP_FAT | GO:0006575—cellular amino acid derivative metabolic process | 14 | 2.857 | 5.97E-04 | BC050378, NM_001032998, BC010082, NM_000236, AJ310724, NM_002084, NM_018208, BC053578, BC108264, NM_018960, NM_000499, NM_001001331, NM_001032364, NM_145740, NM_000787, NM_003937, BC074719, AY310359, NM_199127, J05235, BC069473, BC110891 |
| GOTERM_BP_FAT | GO:0051186—cofactor  metabolic process | 15 | 3.061 | 8.82E-04 | NM_005276, NM_032501, NM_001009185, BC050378, NM_001032998, BC032037, NM_002084, BC029399, BC053578, NM_000613, BC108264, NM_000499, NM_000670, NM_002133, NM_001032364, NM_145740, NM_003937, AY310359, NM_199127, J05235, BC022319, BC069473, BC110891 |
| **Induced Genes after 24 h of Treatment with 5 μM CC1** | | | | | |
| **Category** | **Term** | **Count** | **%** | ***p*-Value** | **Genes** |
| GOTERM_BP_FAT | GO:0042592—homeostatic process | 5 | 13.51 | 0.02649 | NM_005951, NM_001124, NM_005953, BC007034, BC008408, NM_001724, NM_003807 |
| GOTERM_BP_FAT | GO:0009409—response to cold | 2 | 5.405 | 0.03212 | BC004490, NM_001124 |

**Table S1.** *Cont.*

| **Induced Genes after 24 h of Treatment with 5 μM CC1** | | | | | |
| --- | --- | --- | --- | --- | --- |
| **Category** | **Term** | **Count** | **%** | ***p*-Value** | **Genes** |
| GOTERM_BP_FAT | GO:000761—behavior | 4 | 10.81 | 0.03463 | BC004490, NM_001964, NM_002517, BC073983, BC077725 |
| GOTERM_BP_FAT | GO:0048545—response to steroid hormone stimulus | 3 | 8.108 | 0.03526 | BC004490, NM_001124, BC077725 |
| GOTERM_BP_FAT | GO:0010035—response to  inorganic substance | 3 | 8.108 | 0.03973 | BC004490, NM_005952, NM_005951, BC032131, BC008408, BC018190 |
| GOTERM_BP_FAT | GO:0009991—response to  extracellular stimulus | 3 | 8.108 | 0.04514 | BC004490, NM_001124, AY858838 |
| GOTERM_BP_FAT | GO:0030510—regulation of BMP signaling pathway | 2 | 5.405 | 0.04558 | NM_000522, NM_002775 |
| GOTERM_BP_FAT | GO:0030005—cellular di-, tri-valent inorganic cation homeostasis | 3 | 8.108 | 0.04776 | NM_005951, NM_001124, NM_005953, BC007034, BC008408 |

**Table S2. Biological processes affected in HepG2 cells after 10 µM crambescin-C1 (CC1) treatment, as determined by ontology analysis.**

| **Repressed Genes after 24 h of Treatment with 10 μM CC1** | | | | | |
| --- | --- | --- | --- | --- | --- |
| **Category** | **Term** | **Count** | **%** | ***p*-Value** | **Genes** |
| GOTERM_BP_FAT | GO:0000279—M phase | 20 | 4.05679513 | 5.04E-04 | AY927772, NM_052961, BC006510, AB102716, NM_032997, AK125219, AB113249, AK057276, BC034607, NM_006342, NM_018068, AY971957, NM_014750, NM_018063, AF319573, BC111751, AB102720, NM_003686, AB154416, NM_024511, NM_017785, NM_017760, NM_001813, BC001531, NM_018136, NM_004856, NM_002417, Z15005, NM_018365, NM_031966, NM_020238 |
| GOTERM_BP_FAT | GO:0002673—regulation of acute inflammatory response | 5 | 1.01419878 | 0.00153199 | BC022256, NM_000505, BC012350, NM_001879, NM_000892 |
| GOTERM_BP_FAT | GO:0030193—regulation of  blood coagulation | 6 | 1.21703854 | 0.00174283 | NM_000505, NM_000301, NM_002658, BC060039, CR605456, NM_000893, BC013575, NM_001955, NM_000892 |
| GOTERM_BP_FAT | GO:0006260—DNA replication | 13 | 2.63691684 | 0.00257571 | NM_181558, BC020729, NM_021067, BC007015, NM_007370, NM_016448, AF297866, NM_002689, AK024077, NM_022770, NM_032336, BC113116, AK023974, BC014437, BC001531, NM_016095, NM_022111, BC031061 |
| GOTERM_BP_FAT | GO:0042730—fibrinolysis | 4 | 0.81135903 | 0.00273323 | NM_000505, NM_000301, NM_002658, BC013575, NM_000892 |
| GOTERM_BP_FAT | GO:0000280—nuclear division | 14 | 2.83975659 | 0.00310591 | AF319573, AY927772, AB102720, AB154416, NM_024511, AB102716, BC006510, NM_017785, NM_032997, AB113249, AK125219, NM_017760, BC034607, NM_001813, NM_018136, BC001531, AY971957, NM_004856, NM_014750, NM_018063, Z15005, NM_031966, NM_020238 |
| GOTERM_BP_FAT | GO:0007067—mitosis | 14 | 2.83975659 | 0.00310591 | AF319573, AY927772, AB102720, AB154416, NM_024511, AB102716, BC006510, NM_017785, NM_032997, AB113249, AK125219, NM_017760, BC034607, NM_001813, NM_018136, BC001531, AY971957, NM_004856, NM_014750, NM_018063, Z15005, NM_031966, NM_020238 |
| GOTERM_BP_FAT | GO:0050818—regulation  of coagulation | 6 | 1.21703854 | 0.00313522 | NM_000505, NM_000301, NM_002658, BC060039, CR605456, NM_000893, BC013575, NM_001955, NM_000892 |
| GOTERM_BP_FAT | GO:0051919—positive regulation of fibrinolysis | 3 | 0.60851927 | 0.00348701 | NM_000505, NM_000301, NM_000892 |

**Table S2.** *Cont.*

| **Repressed Genes after 24 h of Treatment with 10 μM CC1** | | | | | |
| --- | --- | --- | --- | --- | --- |
| **Category** | **Term** | **Count** | **%** | ***p*-Value** | **Genes** |
| GOTERM_BP_FAT | GO:0000087—M phase of  mitotic cell cycle | 14 | 2.83975659 | 0.00361924 | AF319573, AY927772, AB102720, AB154416, NM_024511, AB102716, BC006510, NM_017785, NM_032997, AB113249, AK125219, NM_017760, BC034607, NM_001813, NM_018136, BC001531, AY971957, NM_004856, NM_014750, NM_018063, Z15005, NM_031966, NM_020238 |
| GOTERM_BP_FAT | GO:0055114—oxidation reduction | 28 | 5.67951318 | 0.00427275 | NM_005276, NM_004109, NM_015718, BC088356, BC029057, BC033009, BC074738, NM_000667, NM_000765, BC074771, NM_000860, BC067419, BC074770, AK092439, NM_000670, NM_018068, BC108742, BC101777, BC101631, NM_023078, AF095737, BC022319, NM_001917, BC111751, NM_017545, NM_000780, NM_005989, BC012027, BC114473, NM_000672, NM_004179, NM_021232, NM_022568, BC067441, NM_000777, BC033862, NM_013387, NM_024955, BC067424, AK058142, BC039065 |
| GOTERM_BP_FAT | GO:0048285—organelle fission | 14 | 2.83975659 | 0.00435079 | AF319573, AY927772, AB102720, AB154416, NM_024511, AB102716, BC006510, NM_017785, NM_032997, AB113249, AK125219, NM_017760, BC034607, NM_001813, NM_018136, BC001531, AY971957, NM_004856, NM_014750, NM_018063, Z15005, NM_031966, NM_020238 |
| GOTERM_BP_FAT | GO:0006081—cellular aldehyde metabolic process | 5 | 1.01419878 | 0.00457943 | NM_022568, NM_001917, BC074770, AF191687, NM_000670, NM_017545, BC029057, BC114473, BC022319 |
| GOTERM_BP_FAT | GO:0000278—mitotic cell cycle | 19 | 3.85395538 | 0.00459511 | AY927772, BC006510, AB102716, NM_032997, AK125219, AB113249, BC034607, NM_005983, AK124863, AY971957, NM_001262, NM_014750, NM_018063, AF319573, AB102720, NM_032637, AB154416, NM_024511, NM_017785, BC065513, NM_017760, NM_001813, NM_003590, BC001531, NM_018136, BC007441, NM_004856, Z15005, NM_031966, NM_020238 |
| GOTERM_BP_FAT | GO:0050000—chromosome localization | 4 | 0.81135903 | 0.00535264 | NM_001813, AY927772, AB154416, NM_014750, Z15005, NM_017785 |
| GOTERM_BP_FAT | GO:0051303—establishment of chromosome localization | 4 | 0.81135903 | 0.00535264 | NM_001813, AY927772, AB154416, NM_014750, Z15005, NM_017785 |
| GOTERM_BP_FAT | GO:0006069—ethanol oxidation | 3 | 0.60851927 | 0.00571753 | BC074771, BC067419, NM_000670, BC033009, NM_000672, BC022319, BC039065, BC074738, NM_000667 |

**Table S2.** *Cont.*

| **Repressed Genes after 24 h of Treatment with 10 μM CC1** | | | | | |
| --- | --- | --- | --- | --- | --- |
| **Category** | **Term** | **Count** | **%** | ***p*-Value** | **Genes** |
| GOTERM_BP_FAT | GO:0006067—ethanol metabolic process | 3 | 0.60851927 | 0.00571753 | BC074771, BC067419, NM_000670, BC033009, NM_000672, BC022319, BC039065, BC074738, NM_000667 |
| GOTERM_BP_FAT | GO:0034308—monohydric alcohol metabolic process | 3 | 0.60851927 | 0.00571753 | BC074771, BC067419, NM_000670, BC033009, NM_000672, BC022319, BC039065, BC074738, NM_000667 |
| GOTERM_BP_FAT | GO:0032101—regulation of response to external stimulus | 11 | 2.23123732 | 0.00587908 | NM_000505, BC060039, NM_002658, BC012350, NM_013372, BC039902, BC022256, NM_000301, CR605456, NM_021804, NM_000893, BC013575, NM_001879, NM_000892, NM_001955 |
| GOTERM_BP_FAT | GO:0046487—glyoxylate  metabolic process | 3 | 0.60851927 | 0.00843765 | NM_001917, BC074770, AF191687, NM_017545, BC029057 |
| GOTERM_BP_FAT | GO:0070613—regulation of protein processing | 3 | 0.60851927 | 0.00843765 | NM_000505, NM_001879, NM_000892 |
| GOTERM_BP_FAT | GO:0010953—regulation of protein maturation by peptide bond cleavage | 3 | 0.60851927 | 0.00843765 | NM_000505, NM_001879, NM_000892 |
| **Induced Genes after 24 h of Treatment with 10 μM CC1** | | | | | |
| **Category** | **Term** | **Count** | **%** | ***p*-Value** | **Genes** |
| GOTERM_BP_ALL | GO:0006694—steroid  biosynthetic process | 10 | 2.79329609 | 1.95E-05 | AB208789, NM_001017369, BC007068, BC050427, BC033692, BC000054, NM_001124, NM_002130, NM_000786, BC010653, NM_004508, NM_000859, NM_001360, BC107879 |
| GOTERM_BP_ALL | GO:0016126—sterol  biosynthetic process | 7 | 1.95530726 | 3.14E-05 | NM_000786, NM_002130, NM_001017369, BC010653, NM_000859, NM_004508, BC050427, BC033692, NM_001360, BC000054, BC107879 |
| GOTERM_BP_ALL | GO:0008610—lipid  biosynthetic process | 18 | 5.02793296 | 6.74E-05 | AK090444, AB208789, CR610092, BC001305, NM_001017369, BC050427, BC011913, NM_001012727, NM_006412, NM_000786, BC010653, BC107879, NM_024090, NM_206918, NM_017436, NM_000954, BC007068, BC033692, BC000054, AY847299, NM_001124, NM_002130, AY847301, NM_003358, NM_024830, NM_000859, NM_004508, NM_001360 |
| GOTERM_BP_ALL | GO:0001666—response to hypoxia | 11 | 3.0726257 | 1.40E-04 | AF064599, NM_000930, NM_002608, NM_005165, X83705, BC106925, AF323587, AK123350, BC029822, NM_002226, NM_001039667, NM_001124, NM_003946, NM_001216, BC077725, AK126941, NM_022073 |

**Table S2.** *Cont.*

| **Induced Genes after 24 h of Treatment with 10 μM CC1** | | | | | |
| --- | --- | --- | --- | --- | --- |
| **Category** | **Term** | **Count** | **%** | ***p*-Value** | **Genes** |
| GOTERM_BP_ALL | GO:0070482—response to  oxygen levels | 11 | 3.0726257 | 2.12E-04 | AF064599, NM_000930, NM_002608, NM_005165, X83705, BC106925, AF323587, AK123350, BC029822, NM_002226, NM_001039667, NM_001124, NM_003946, NM_001216, BC077725, AK126941, NM_022073 |
| GOTERM_BP_ALL | GO:0006066—alcohol  metabolic process | 20 | 5.58659218 | 2.47E-04 | NM_002301, BC043388, NM_000188, AF056320, NM_017448, NM_001017369, BC050540, BC050427, NM_000665, BC032865, NM_024607, AY313926, AY313927, NM_001030287, NM_000786, BC105060, AF334270, BC010653, AK131307, BC107879, BC094752, NM_004566, NM_004567, NM_000952, NM_005165, BC033692, BC106925, BC000054, NM_015978, NM_001003680, NM_002130, BC012625, NM_000859, NM_004508, NM_001360, AB078026, S52624 |
| GOTERM_BP_ALL | GO:0042221—response to  chemical stimulus | 40 | 11.1731844 | 6.09E-04 | BC051726, NM_003304, NM_003280, BC032131, NM_006785, X83705, NM_002773, NM_005252, NM_000602, BC018190, NM_005952, NM_005951, NM_001216, NM_000952, BC106925, NM_012212, AK123350, BC029822, NM_001039667, NM_001124, NM_001003680, BC039894, BC008674, NM_001958, NM_022073, AK126941, S52624, NM_003225, NM_173199, NM_004417, AF323587, BC030811, BC011913, D78579, U31110, NM_002226, NM_002229, NM_002228, NM_003946, NM_006033, NM_014584, BC004490, AK027734, BC014064, AF064599, NM_000930, NM_000164, BC030244, NM_002608, NM_005165, BC030143, BC030537, NM_018155, AY847299, NM_014424, NM_002130, AY847301, BC008408, BC032811, NM_145693, BC077725, NM_004235 |
| GOTERM_BP_ALL | GO:0006695—cholesterol  biosynthetic process | 5 | 1.39664804 | 0.00107224 | NM_000786, NM_002130, NM_000859, NM_004508, BC033692, NM_001360, BC000054 |
| GOTERM_BP_ALL | GO:0010035—response to  inorganic substance | 12 | 3.35195531 | 0.00111989 | BC004490, NM_003225, NM_004417, NM_003304, BC030244, NM_003280, BC032131, NM_005252, NM_000602, BC018190, NM_005952, U31110, AY847299, NM_005951, BC011913, NM_002228, BC039894, AY847301, BC032811, BC008408, NM_001958 |
| GOTERM_BP_ALL | GO:0016125—sterol  metabolic process | 8 | 2.23463687 | 0.00215149 | NM_001003680, NM_000786, NM_002130, NM_001017369, BC010653, NM_000859, NM_004508, BC050427, BC033692, NM_001360, BC000054, BC107879 |

**Table S2.** *Cont.*

| **Induced Genes after 24 h of Treatment with 10 μM CC1** | | | | | |
| --- | --- | --- | --- | --- | --- |
| **Category** | **Term** | **Count** | **%** | ***p*-Value** | **Genes** |
| GOTERM_BP_ALL | GO:0045941—positive regulation  of transcription | 21 | 5.86592179 | 0.00264114 | NM_173199, NM_003709, BC012919, NM_002135, NM_005252, NM_002943, AF323587, BC030811, NM_004821, D78579, NM_002229, NM_002228, AB033012, NM_013376, NM_005461, NM_017521, BC004490, AK027734, NM_004405, BC032558, BC071983, BX537890, AF294278, NM_005239, NM_001538, NM_016270, BC032148, NM_004235, AB078876 |
| GOTERM_BP_ALL | GO:0008202—steroid  metabolic process | 11 | 3.0726257 | 0.0033505 | AB208789, NM_001017369, BC007068, BC050427, BC033692, BC000054, NM_001124, NM_001003680, NM_002130, NM_000786, BC010653, NM_004508, NM_000859, NM_001360, BC107879 |
| GOTERM_BP_ALL | GO:0010628—positive regulation of gene expression | 21 | 5.86592179 | 0.00372028 | NM_173199, NM_003709, BC012919, NM_002135, NM_005252, NM_002943, AF323587, BC030811, NM_004821, D78579, NM_002229, NM_002228, AB033012, NM_013376, NM_005461, NM_017521, BC004490, AK027734, NM_004405, BC032558, BC071983, BX537890, AF294278, NM_005239, NM_001538, NM_016270, BC032148, NM_004235, AB078876 |
| GOTERM_BP_ALL | GO:0045935—positive regulation of nucleobase, nucleoside, nucleotide and nucleic acid metabolic process | 22 | 6.1452514 | 0.00386118 | NM_173199, NM_003709, BC012919, X83705, NM_005252, NM_002135, AF323587, NM_002943, BC030811, NM_004821, D78579, NM_002229, NM_002228, AB033012, NM_013376, NM_005461, NM_017521, BC004490, AK027734, NM_004405, BC032558, BC071983, BX537890, NM_002608, AF294278, BC029822, NM_005239, NM_001538, NM_016270, BC077725, BC032148, NM_004235, AB078876 |
| GOTERM_BP_ALL | GO:0051239—regulation of multicellular organismal process | 29 | 8.10055866 | 0.00483419 | NM_003407, CR610092, NM_003280, NM_006785, X83705, AF323587, NM_000602, NM_000665, NM_001731, BC011913, BC000013, NM_000598, NM_001012727, NM_002228, NM_006412, BC105060, AF334270, NM_006033, NM_005461, BC109265, NM_004405, AK027734, BC015749, BC094752, BC032558, NM_000930, BC030244, NM_177551, NM_002608, NM_000954, BC030143, AF294278, NM_023004, NM_003565, BC029822, AY847299, NM_001039667, NM_001124, NM_021649, NM_014424, AY847301, NM_006018, BC077725, AK126941, NM_001005407, AB078876 |
| GOTERM_BP_ALL | GO:0010033—response to  organic substance | 24 | 6.70391061 | 0.00489033 | BC051726, NM_004417, NM_173199, NM_003225, NM_006785, X83705, NM_002773, NM_005252, D78579, BC011913, NM_002229, NM_002228, NM_001216, NM_014584, AK027734, BC014064, BC004490, NM_000952, NM_002608, NM_005165, BC030143, BC030537, BC106925, BC029822, NM_018155, AY847299, NM_001003680, NM_001124, BC039894, NM_014424, NM_002130, BC008674, AY847301, BC032811, NM_145693, BC077725, AK126941, S52624 |

**Table S2.** *Cont.*

| **Induced Genes after 24 h of Treatment with 10 μM CC1** | | | | | |
| --- | --- | --- | --- | --- | --- |
| **Category** | **Term** | **Count** | **%** | ***p*-Value** | **Genes** |
| GOTERM_BP_ALL | GO:0006629—lipid metabolic process | 26 | 7.26256983 | 0.00538564 | AK090444, AB208789, CR610092, BC001305, NM_001017369, BC050427, NM_015715, BC011913, NM_000783, NM_001012727, NM_006412, NM_000786, NM_203347, BC010653, NM_006033, BC107879, BC035124, NM_024090, NM_206918, NM_017436, NM_001037290, NM_000954, BC007068, BC030537, BC033692, NM_012212, BC000054, AY847299, NM_001124, NM_001003680, NM_002130, AY847301, NM_003358, BC093893, NM_024830, NM_145693, NM_000859, NM_004508, NM_001360 |
| GOTERM_BP_ALL | GO:0051173—positive regulation of nitrogen compound metabolic process | 22 | 6.1452514 | 0.00554068 | NM_173199, NM_003709, BC012919, X83705, NM_005252, NM_002135, AF323587, NM_002943, BC030811, NM_004821, D78579, NM_002229, NM_002228, AB033012, NM_013376, NM_005461, NM_017521, BC004490, AK027734, NM_004405, BC032558, BC071983, BX537890, NM_002608, AF294278, BC029822, NM_005239, NM_001538, NM_016270, BC077725, BC032148, NM_004235, AB078876 |
| GOTERM_BP_ALL | GO:0009719—response to  endogenous stimulus | 16 | 4.46927374 | 0.00600529 | NM_173199, NM_003225, NM_004417, X83705, NM_005252, NM_002773, D78579, NM_002229, NM_001216, BC004490, BC014064, AK027734, NM_002608, NM_005165, BC030537, BC106925, BC029822, NM_018155, NM_001003680, NM_001124, BC039894, NM_002130, NM_145693, BC032811, BC077725 |
| GOTERM_BP_ALL | GO:0010557—positive regulation of macromolecule biosynthetic process | 22 | 6.1452514 | 0.00655139 | NM_173199, NM_003709, BC012919, X83705, NM_005252, NM_002135, AF323587, NM_002943, BC030811, NM_004821, D78579, NM_002229, NM_002228, AB033012, NM_013376, NM_005461, NM_017521, BC004490, AK027734, NM_004405, BC032558, BC071983, BX537890, NM_002608, AF294278, BC029822, NM_005239, NM_001538, NM_016270, BC077725, BC032148, NM_004235, AB078876 |
| GOTERM_BP_ALL | GO:0005975—carbohydrate  metabolic process | 19 | 5.30726257 | 0.0066425 | NM_003225, NM_002301, BC043388, NM_000188, BC039196, AF056320, NM_017448, BC050540, BC032865, NM_024607, NM_020469, NM_030765, AY313926, AY313927, NM_001030287, NM_006931, NM_003943, AK131307, BC109101, BC012892, AF502910, BC074885, NM_004566, NM_004567, NM_000952, NM_005165, BC106925, NM_015978, NM_002557, NM_003549, BC012625, BC032811, AB078026, S52624 |

**Table S2.** *Cont.*

| **Induced Genes after 24 h of Treatment with 10 μM CC1** | | | | | |
| --- | --- | --- | --- | --- | --- |
| **Category** | **Term** | **Count** | **%** | ***p*-Value** | **Genes** |
| GOTERM_BP_ALL | GO:0050794—regulation of cellular process | 142 | 39.6648045 | 0.00706715 | NM_014950, NM_004354, NM_001003795, BC018058, NM_000602, NM_001012727, AY313926, NM_000783, NM_001009992, AY313927, BC038856, BC105060, BC021104, NM_014417, NM_032827, NM_006734, NM_018660, BC109265, AK127046, NM_006732, NM_017521, AK000249, AF087853, NM_001002914, AK075540, NM_001015881, NM_005204, AK123350, BC039894, NM_016270, BC012625, BC007012, BC012919, NM_001003940, NM_004331, BC000013, U31110, NM_019105, NM_001004725, NM_006613, BC033571, NM_005461, BC012907, AK027734, BC015749, NM_006989, NM_003867, BC105131, BC069328, NM_005374, NM_002886, NM_004235, NM_001390, AK124948, NM_006785, NM_015557, NM_005860, X83705, NM_001001418, BC044919, Y11593, U70730, BC031405, NM_005195, NM_012098, AF334270, NM_020400, BC094752, BC032558, NM_177551, AK129684, NM_004626, AY358869, NM_000759, NM_003507, NM_001003680, NM_001124, NM_000859, NM_007207, BC110847, NM_022073, BC041709, S52624, BC104805, NM_173199, NM_015675, BC104807, AK026076, BC104983, NM_000665, NM_001731, BC074791, BC011913, NM_002226, NM_002229, BC066334, NM_002228, NM_001030287, NM_001013253, NM_006033, NM_006172, BC104186, BC102029, BC004490, AF064599, NM_153607, NM_000164, BC015915, BC001785, NM_002608, AF294278, BC030537, NM_006943, U73844, NM_016154, AY847299, BC110598, BC008408, BC077725, BC032148, NM_004165, NM_016084, AB033012, NM_001165, NM_013376, NM_002701, BC074922, BC020712, NM_000913, BX537890, AK126012, NM_001039667, NM_004472, BC069505, NM_004669, NM_001538, NM_001176, NM_006018, AK126941, AB078026, NM_178129, NM_003709, NM_003597, NM_002135, BC030811, NM_000598, NM_006412, NM_001015045, NM_006813, NM_138445, NM_203289, BC086875, AF027169, BC001237, BC030244, NM_023004, AB020721, BC030143, BC091504, NM_032594, BC074813, NM_001360, NM_001012426, NM_016215, CR936598, BC018112, NM_024652, NM_004433, NM_003304, NM_003407, NM_003280, NM_005253, NM_005252, NM_002943, NM_004821, NM_001017363, NM_005951, NM_182663, NM_014059, Z11898, NM_000952, BC033670, NM_000954, NM_198827, BC029822, NM_003811, NM_005239, NM_021649, AF217985, BC004564, BC012368, NM_001958, NM_004417, BC032518, BX647768, CR610092, BC012362, BC012361, AF323587, NM_002566, D78579, AB046787, NM_003946, NM_004420, NM_003807, NM_004405, NM_000930, BC071983, BC032502, BC094832, BC038978, NM_005220, BC025261, BC033692, BC000054, NM_033285, NM_003565, AF354656, AY847301, NM_014470, NM_145693, NM_005414, AB078876 |

**Table S2.** *Cont.*

| **Induced Genes after 24 h of Treatment with 10 μM CC1** | | | | | |
| --- | --- | --- | --- | --- | --- |
| **Category** | **Term** | **Count** | **%** | ***p*-Value** | **Genes** |
| GOTERM_BP_ALL | GO:0048545—response to steroid hormone stimulus | 10 | 2.79329609 | 0.007498 | BC014064, BC004490, NM_004417, NM_003225, NM_002608, X83705, NM_002773, NM_005252, BC029822, NM_018155, NM_001124, NM_001003680, NM_002229, NM_001216, BC032811, BC077725 |
| GOTERM_BP_ALL | GO:0019318—hexose  metabolic process | 10 | 2.79329609 | 0.007498 | NM_002301, BC043388, NM_000188, NM_004566, NM_004567, AF056320, NM_017448, NM_005165, BC050540, BC106925, BC032865, NM_024607, NM_015978, AY313926, AY313927, NM_001030287, BC012625, AK131307, AB078026 |
| GOTERM_BP_ALL | GO:0006720—isoprenoid  metabolic process | 5 | 1.39664804 | 0.00758696 | NM_000783, NM_002130, NM_001037290, NM_000859, NM_004508, BC033692 |
| GOTERM_BP_ALL | GO:0048660—regulation of smooth muscle cell proliferation | 5 | 1.39664804 | 0.00887243 | NM_000598, BC000013, NM_002228, NM_002608, X83705, BC077725, AF323587, NM_004235, BC030811, BC029822 |

© 2015 by the authors; licensee MDPI, Basel, Switzerland. This article is an open access article distributed under the terms and conditions of the Creative Commons Attribution license (http://creativecommons.org/licenses/by/4.0/).
